# Supplementary material for: Reference Transcriptomes of Porcine Peripheral Immune Cells Created Through Bulk and Single-Cell RNA Sequencing
Source: Front Genet. 2021 Jun 23;12:689406. doi: 10.3389/fgene.2021.689406 (PMC8261551; doi:10.3389/fgene.2021.689406)
Supplement: Supplementary file 15 [file Table_15.DOCX]

**Supplementary figures**


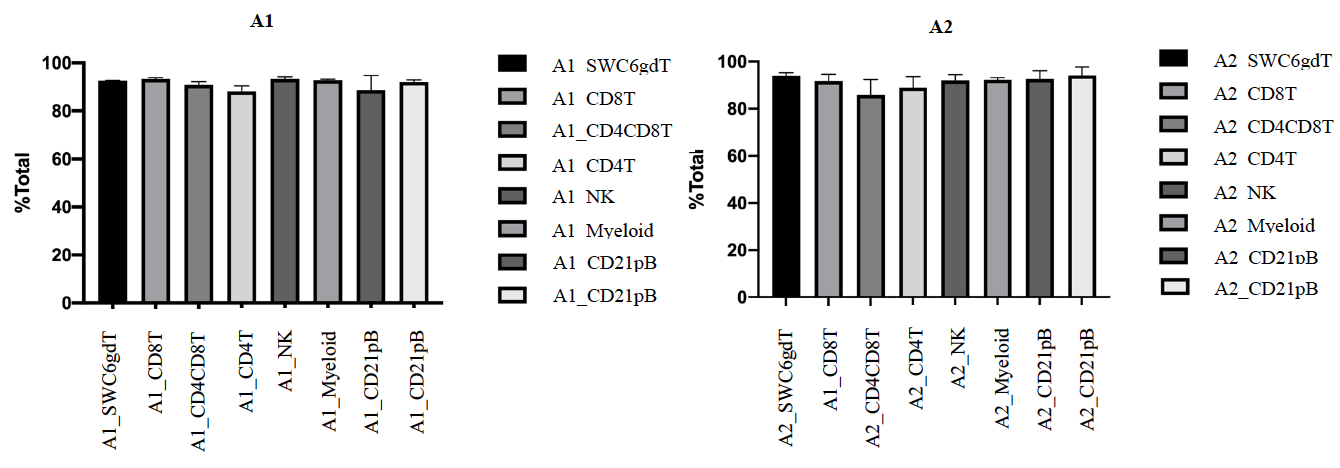


**Supplementary Figure 1.** Percentages for purity yield of sorted cells from two porcine males using BD FACSymphony™.


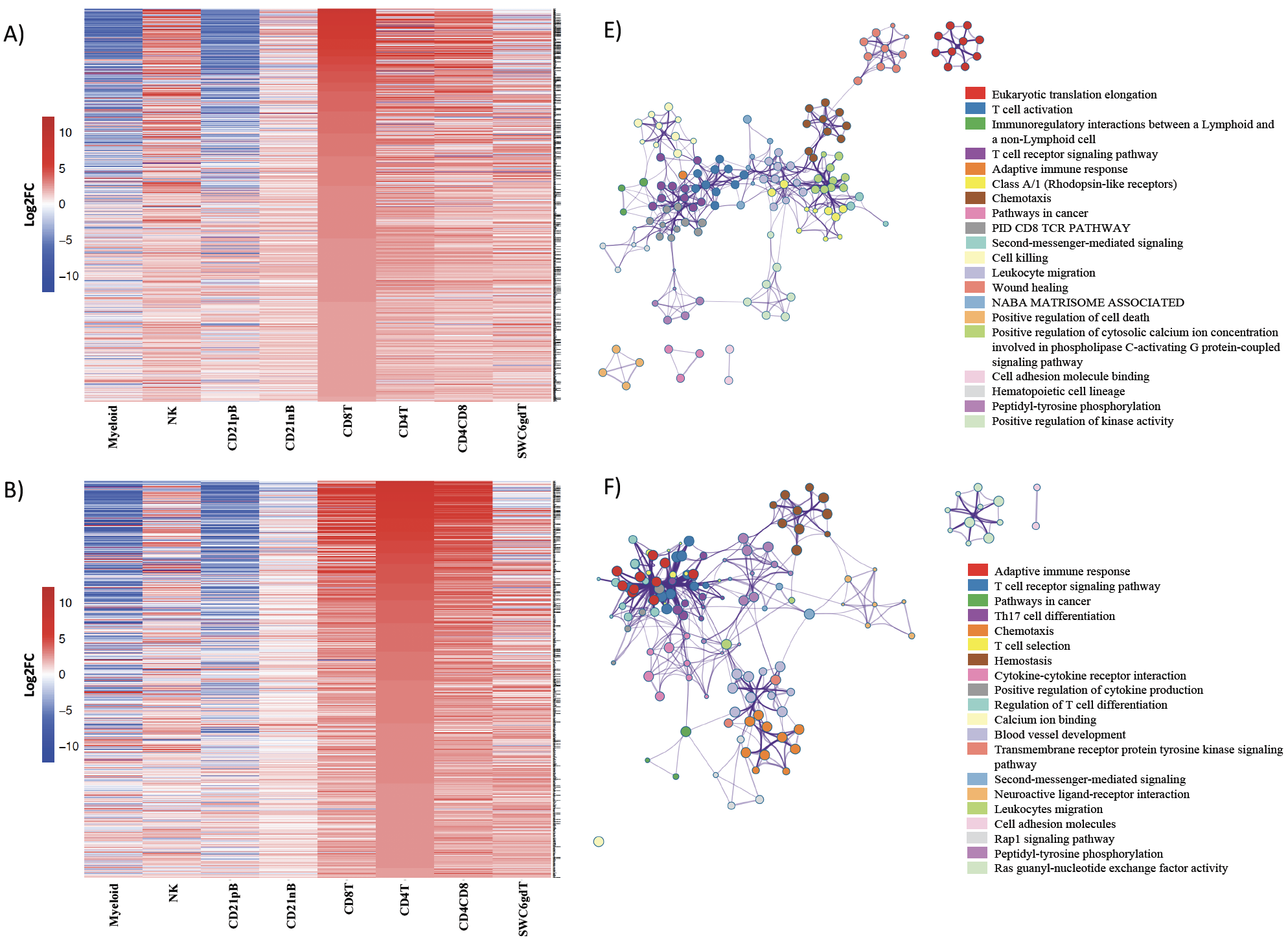

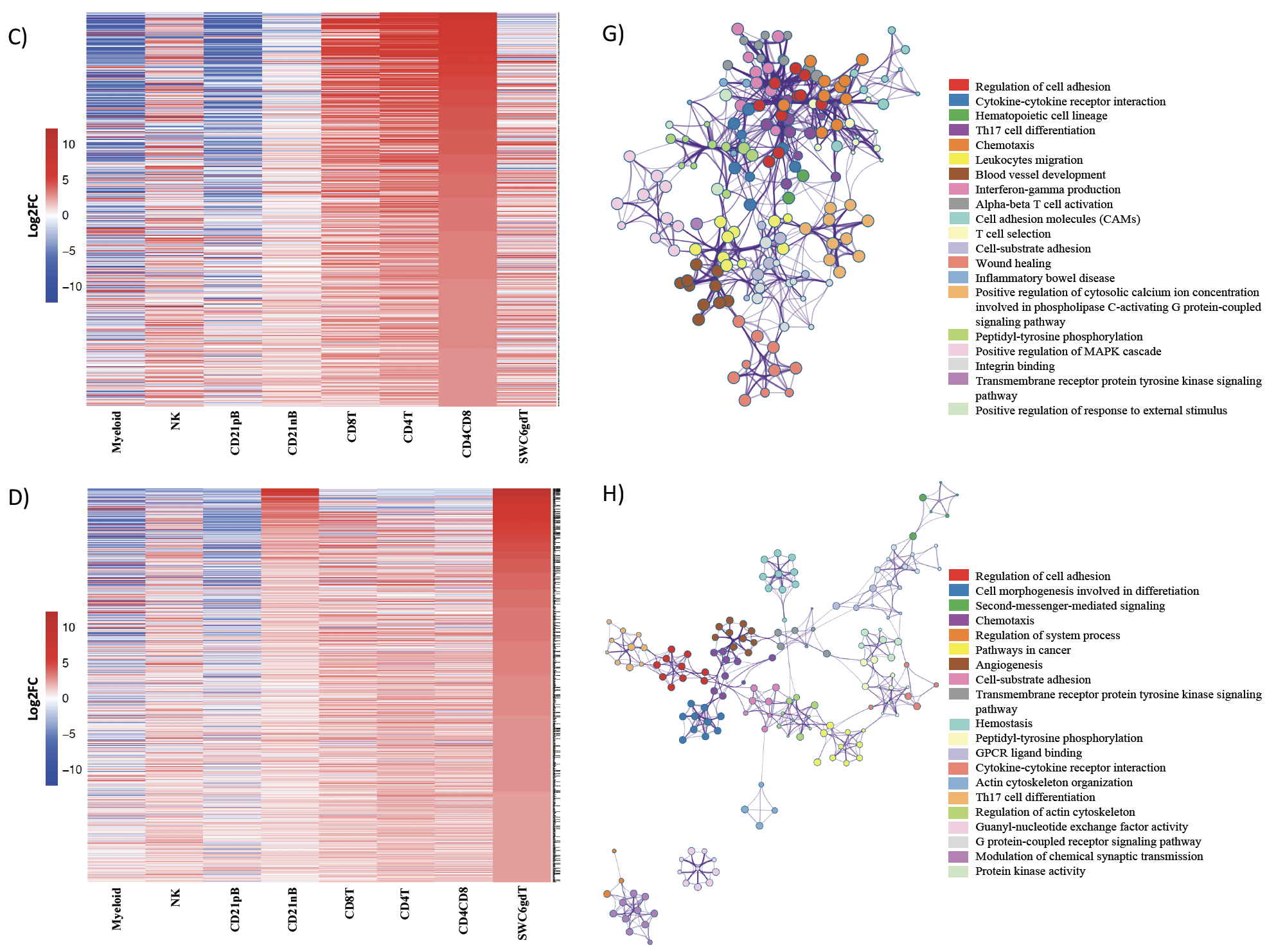


**Supplementary Figure 2.** Top 25% highly enriched genes in CD3^+^ sorted cells. Heatmap showing in decreasing order the top 25% of highly enriched genes in **A**) CD8T, **B**) CD4T, **C**) CD4CD8T and **D**) SWC6gdT cells. Ontology enrichment clusters of the top 25% highly enriched genes of **E**) CD8T, **F**) CD4T, **G**) CD4CD8T and **H**) SWC6gdT cells. The most statistically significant term within similar term clusters was chosen to represent the cluster. Term color is given by cluster ID and the size of the terms is given by –log10 P-value. The stronger the similarity among terms, the

thicker the edges between them.


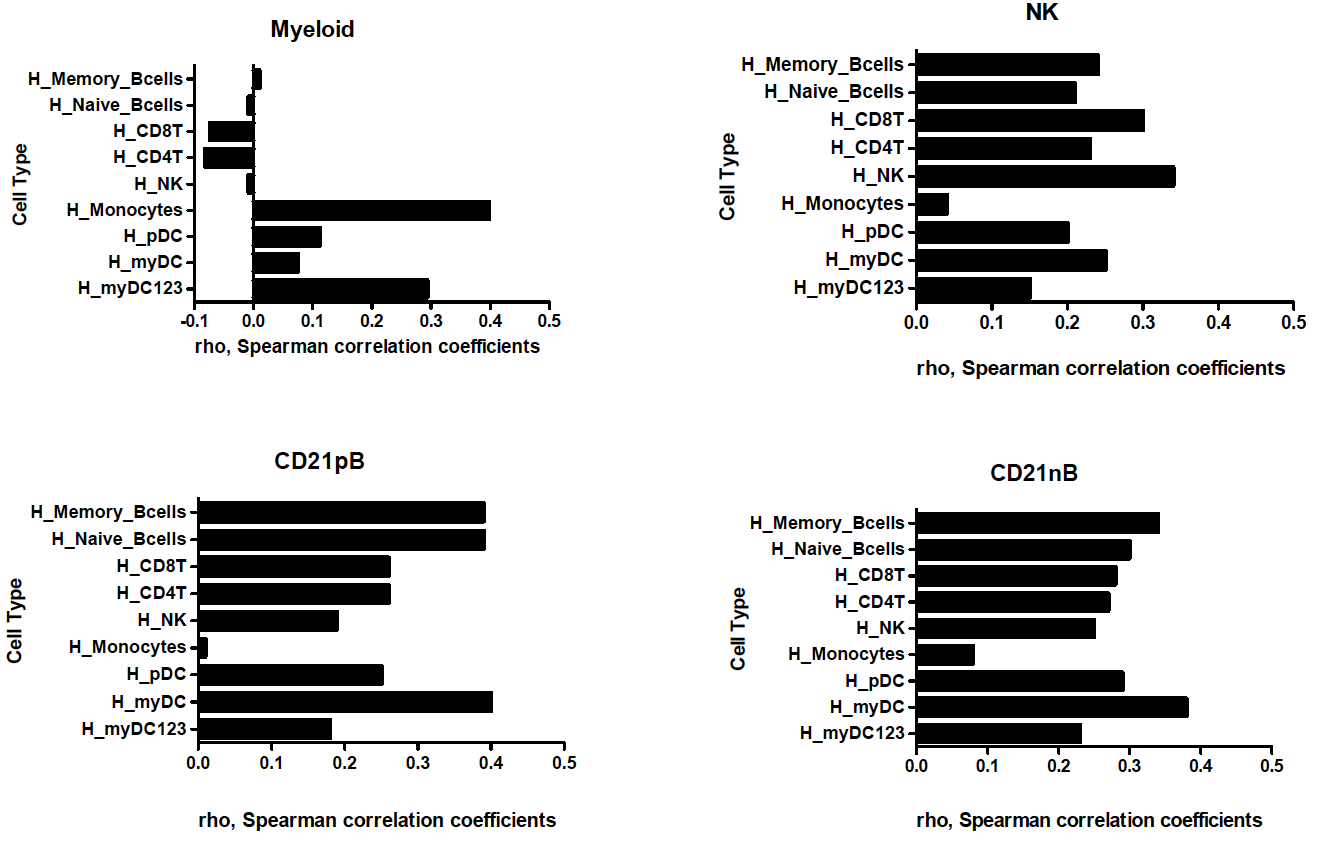


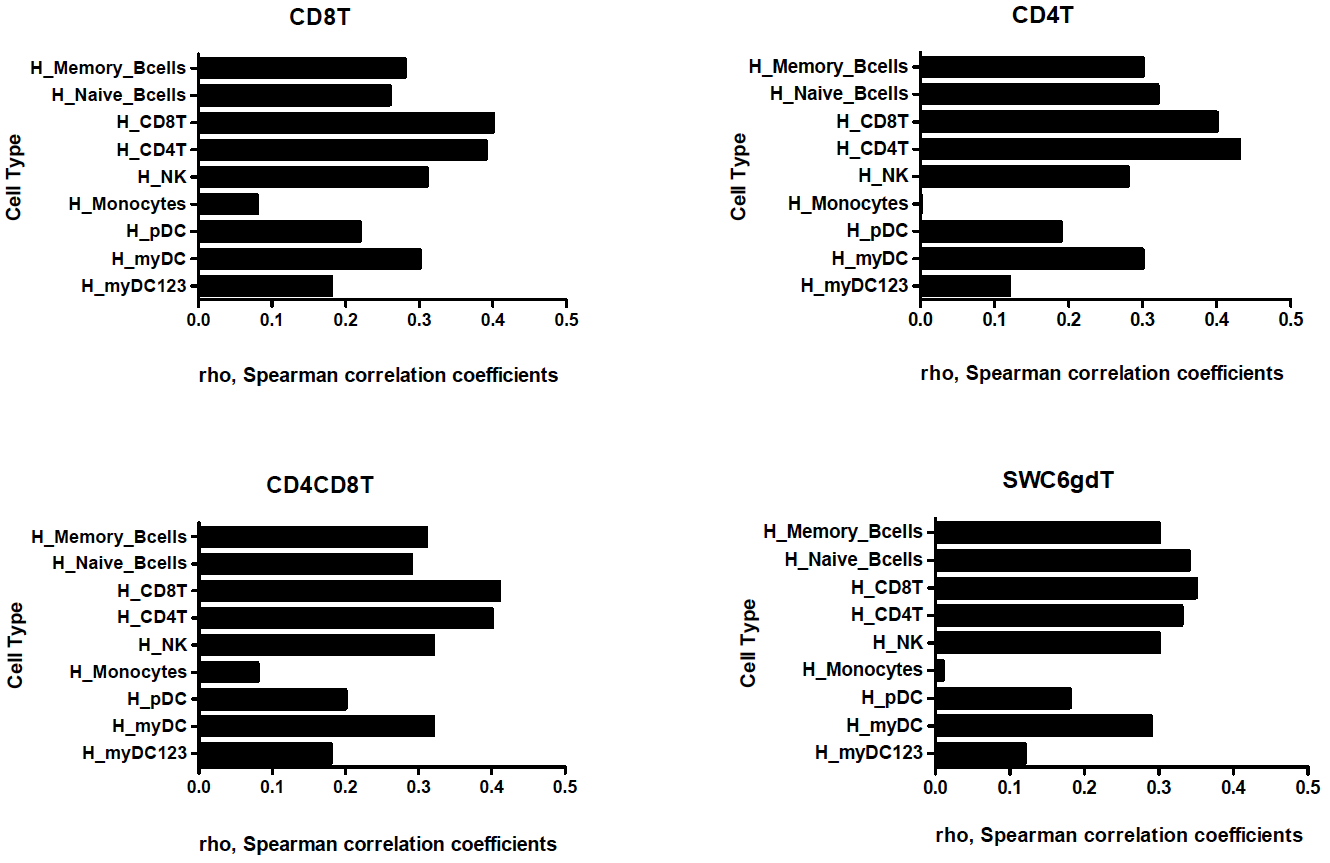


**Supplementary Figure 3.** Spearman rank correlation between TPM values of expressed genes in porcine sorted immune cells and their orthologous genes in human sorted immune cells.


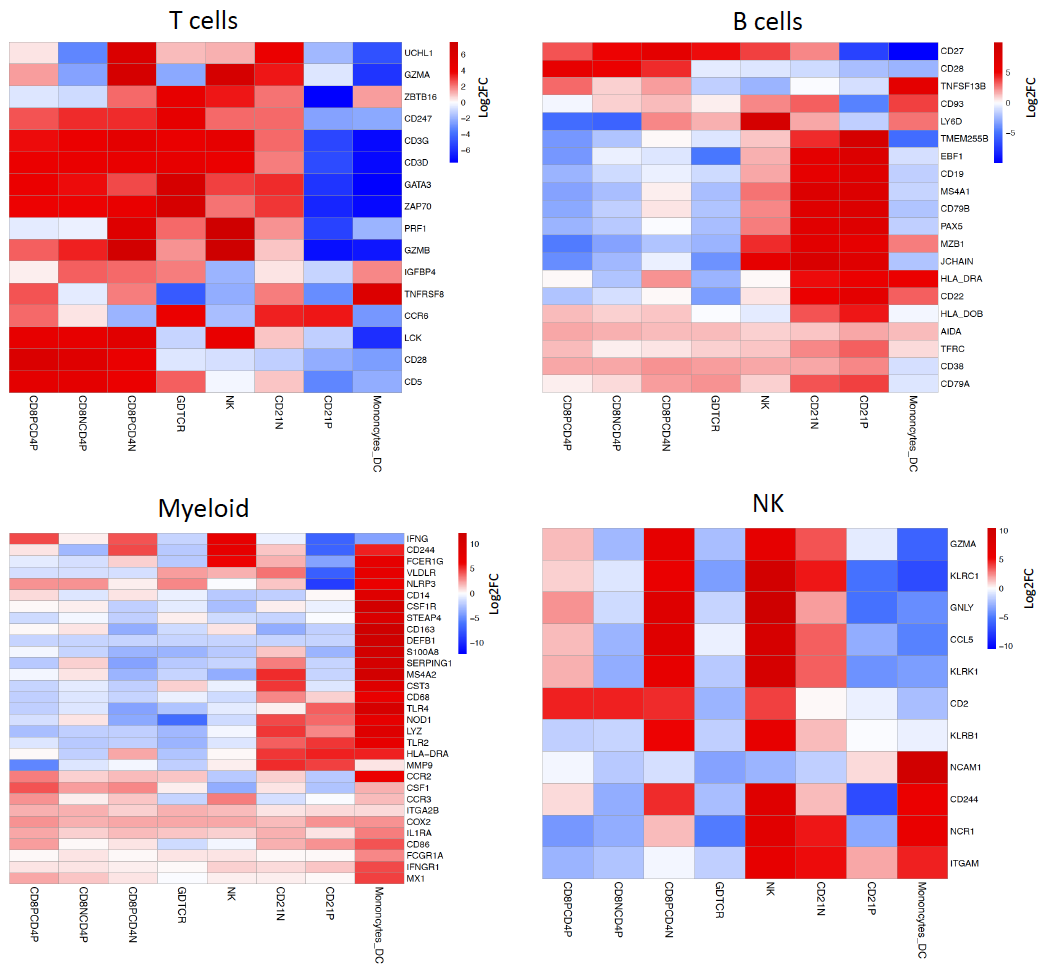


**Supplementary Figure 4.** Gene enrichment heatmap of highly expressed genes and cell markers reported in human and mouse databases in T cells, B cells, myeloid and NK cells.


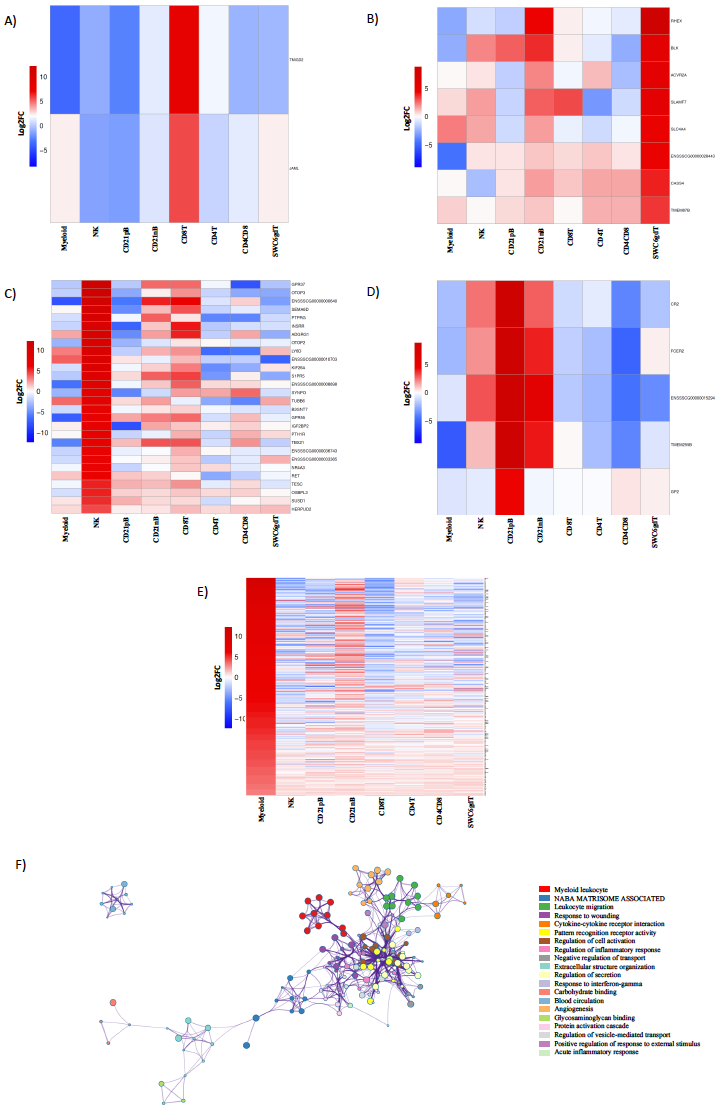


**Supplementary Figure 5.** Cell-type specific genes in CD8T, SWC6gdT, NK, CD21pB and myeloid cells. Heatmap showing in decreasing order the cell-type specific genes in **A**) CD8T, **B**) SWC6gdT, **C**) NK, **D)** CD21pB and **E)** myeloid. Ontology enrichment clusters cell-type specific genes of **F**) myeloid cells. The most statistically significant term within similar term cluster was chosen to represent the cluster. The most statistically significant term within similar term clusters was chosen to represent the cluster. Term color is given by cluster ID and the size of the terms is given by –log10 P-value. The stronger the similarity among terms, the thicker the edges between them.


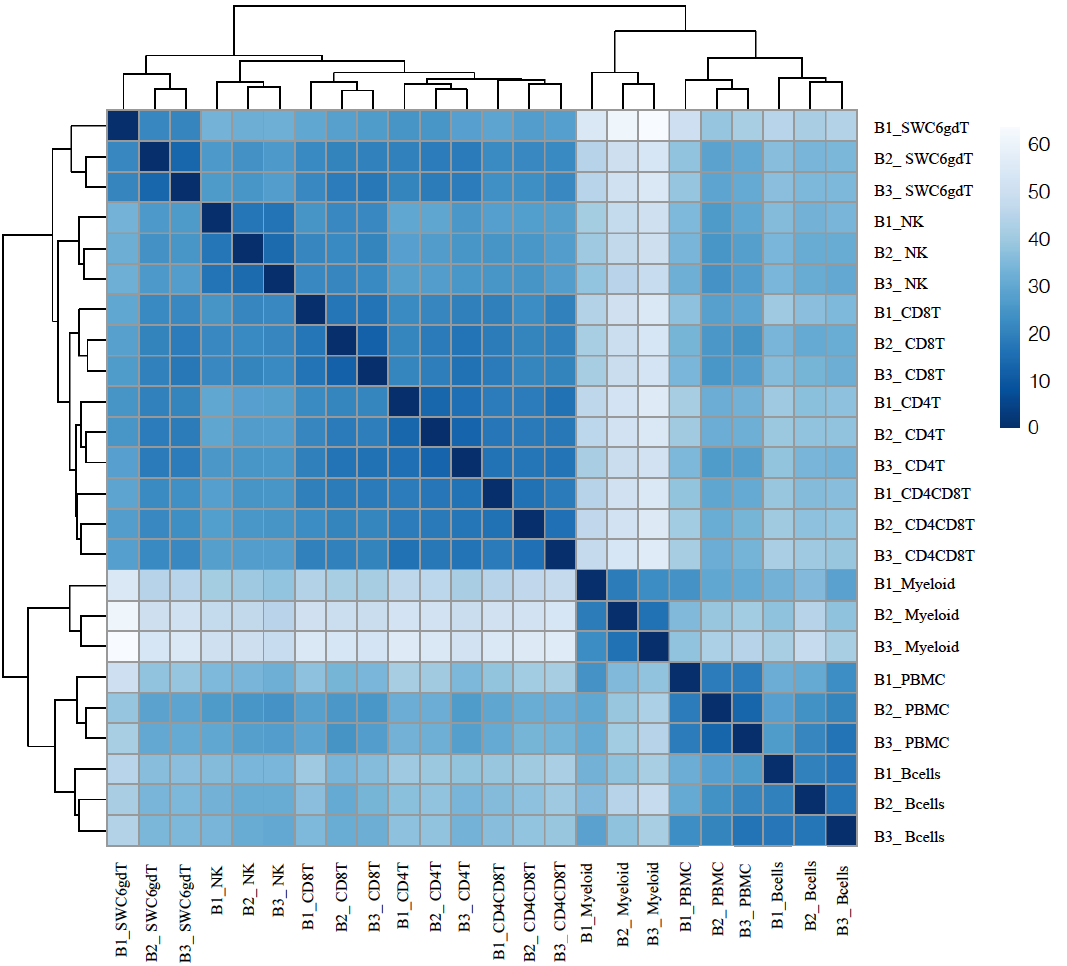


**Supplementary Figure 6.** Gene expression patterns of sorted immune cells showed closer clustering than those share far common progenitors during hematopoiesis. Principal component analysis of transformed reads counts for selected genes in Nanostring. Axis indicate component scores.


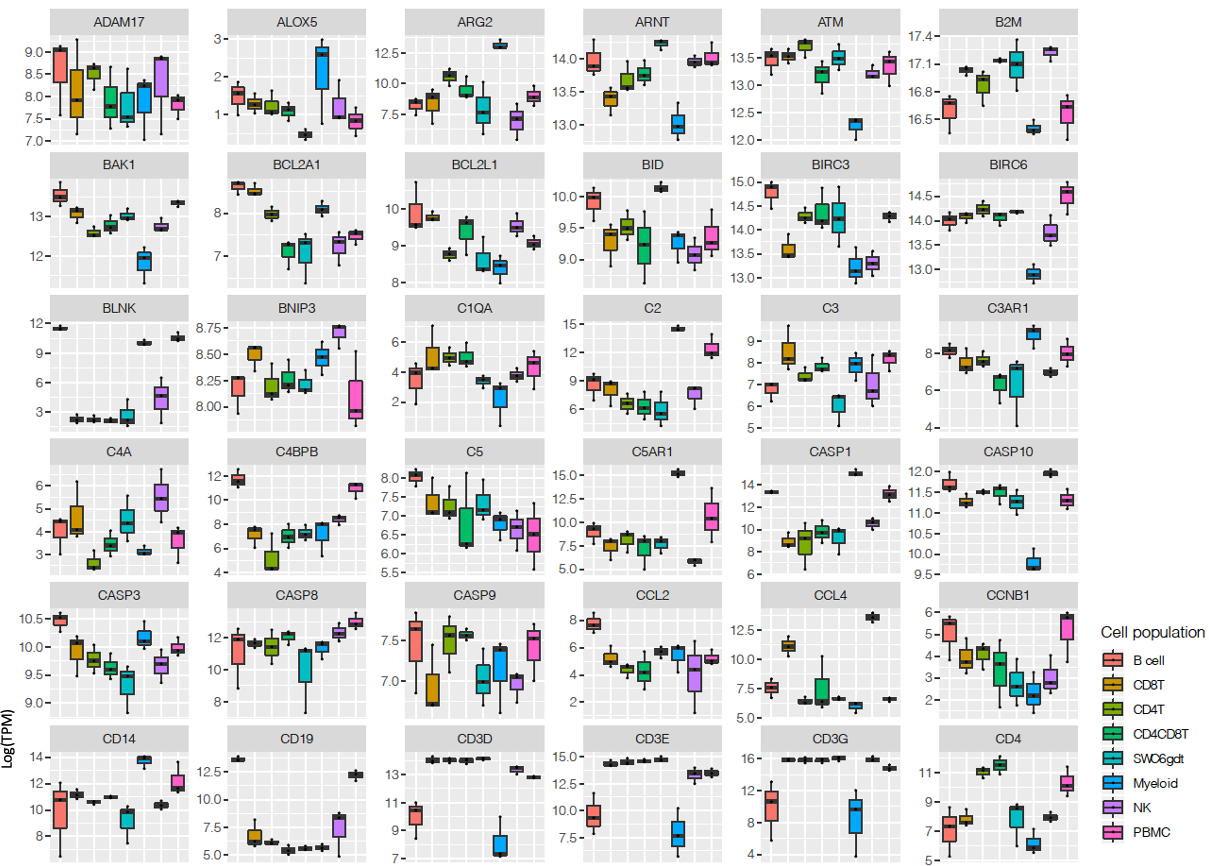


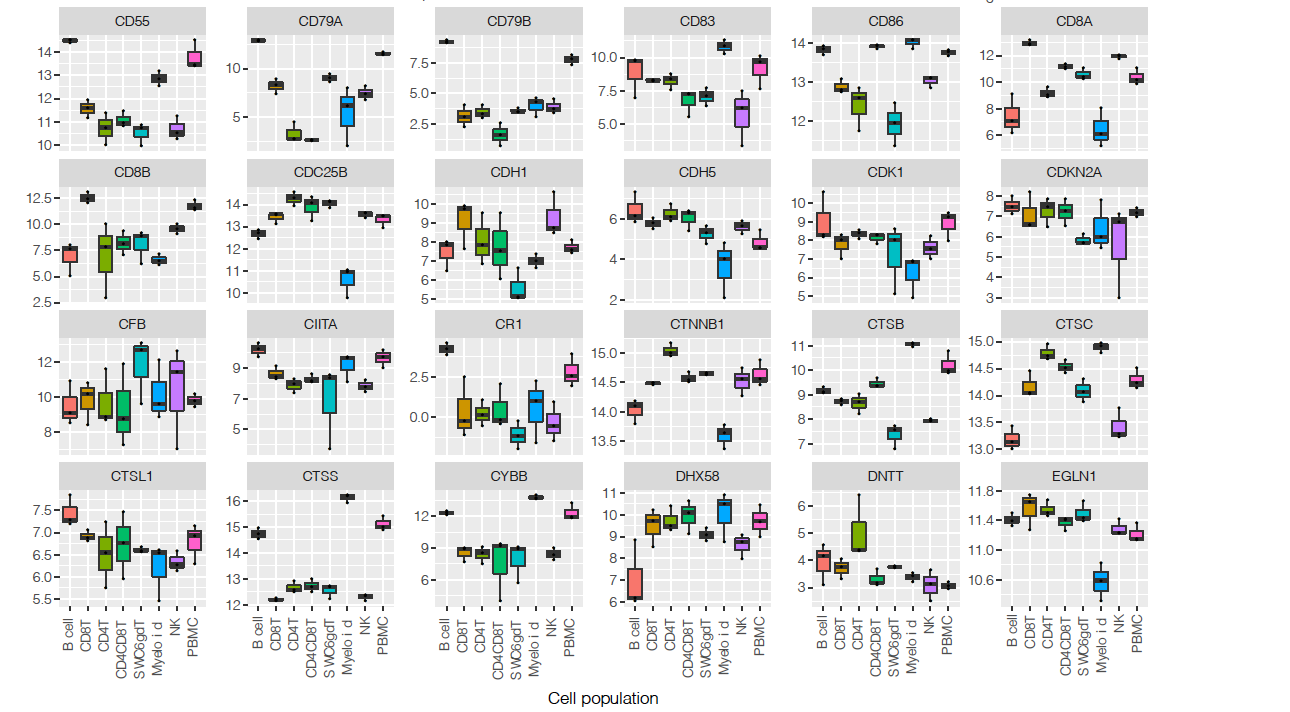


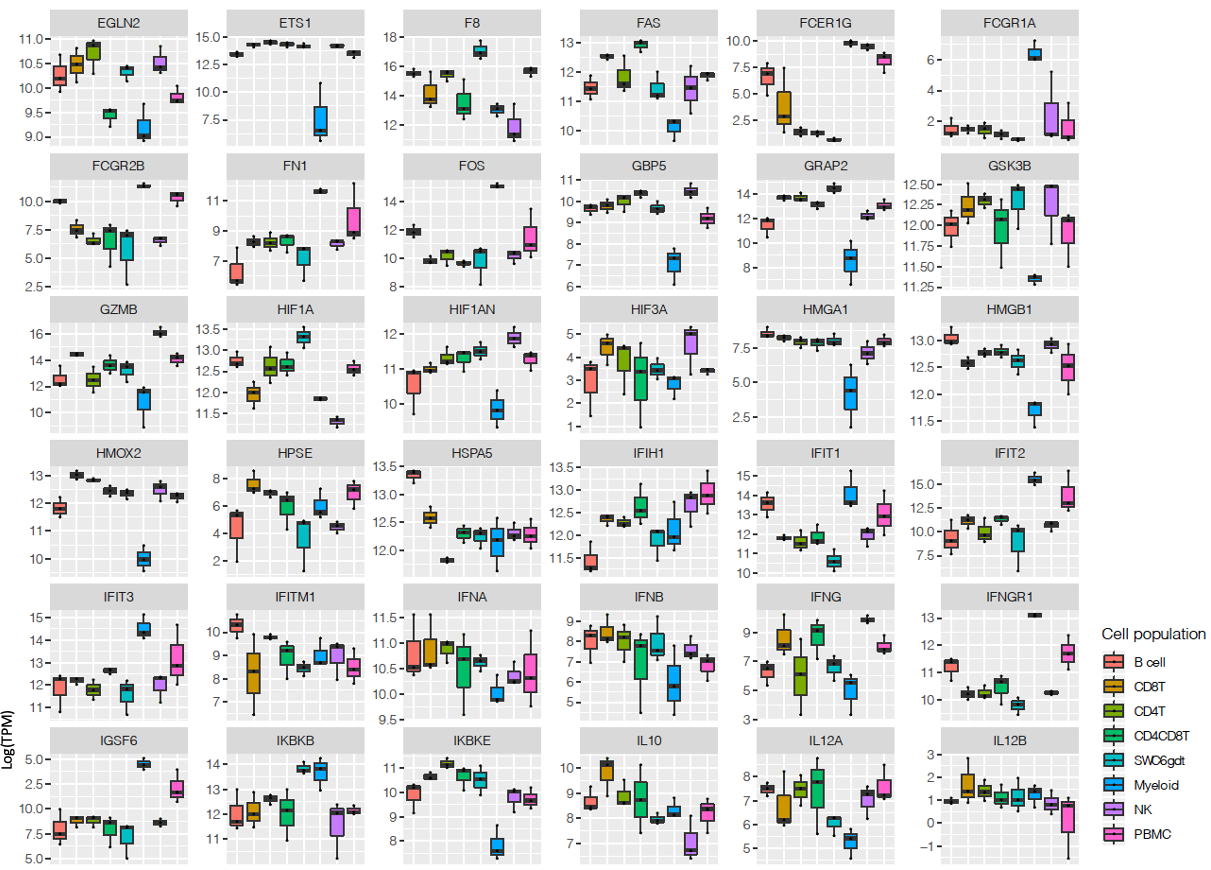


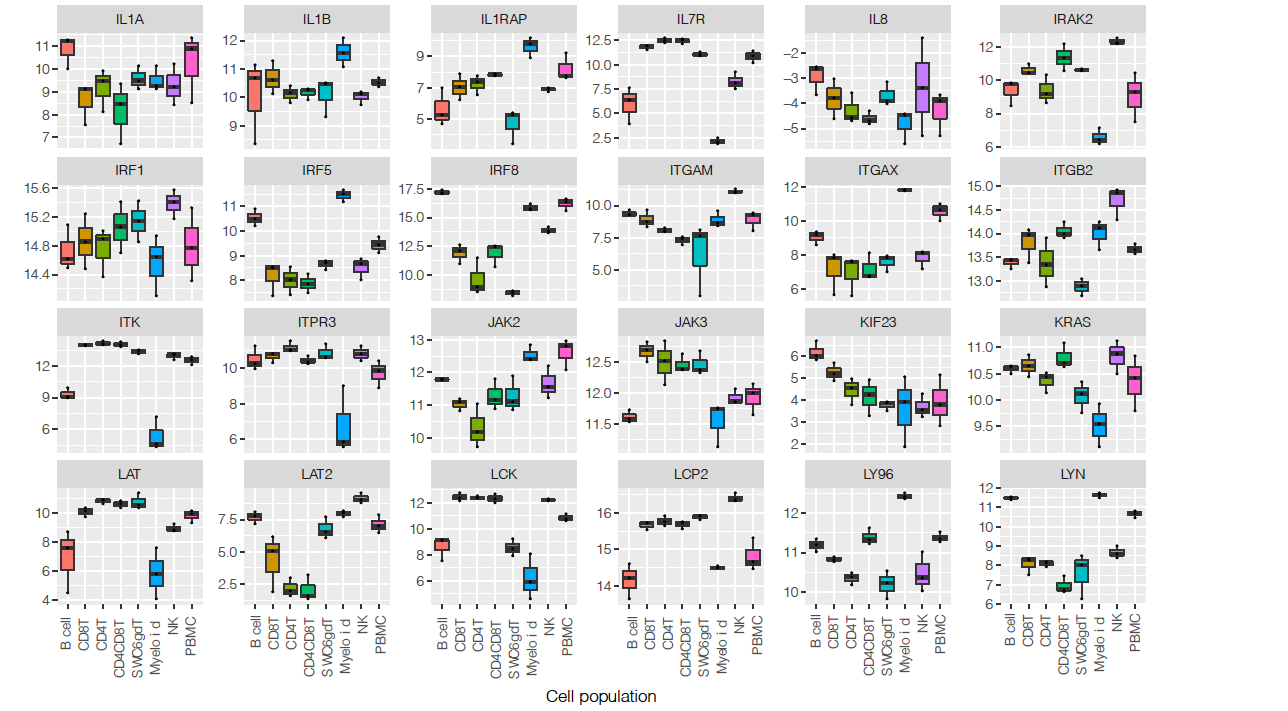


**
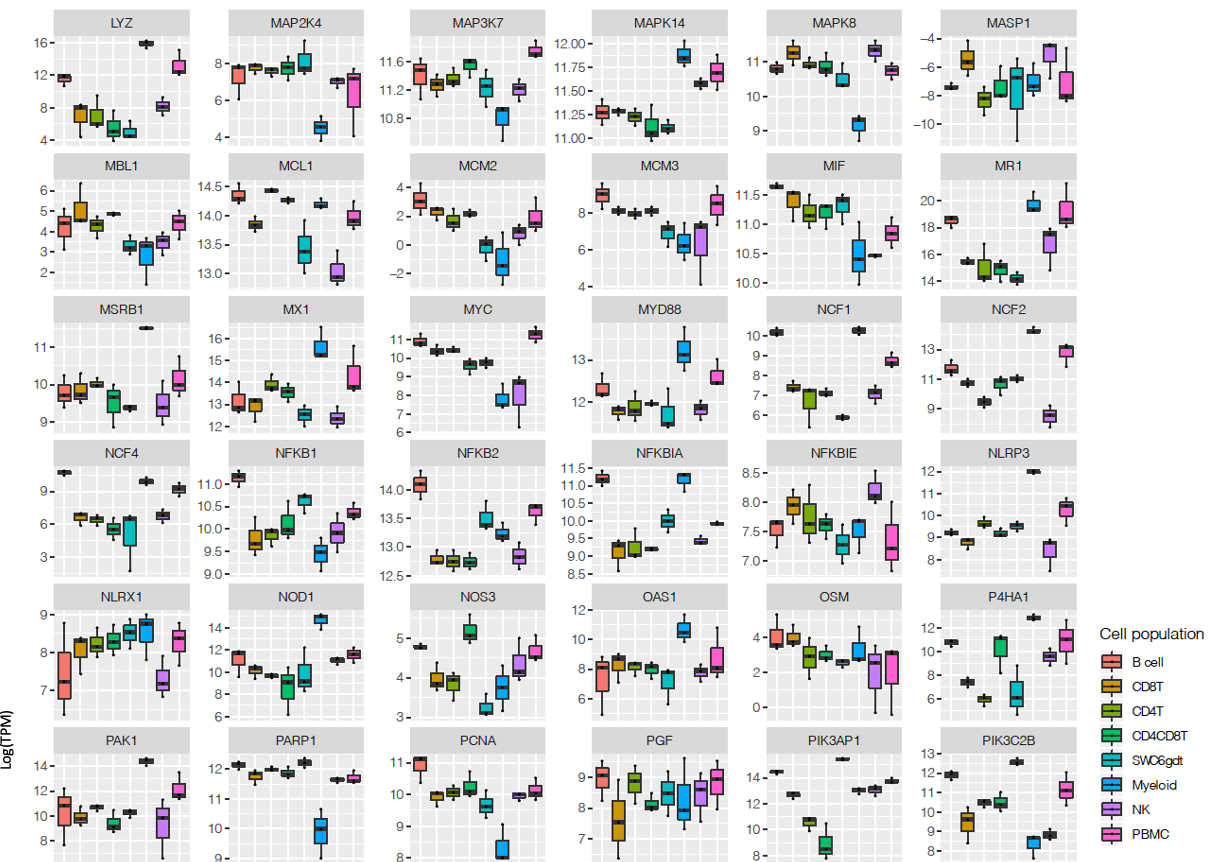
**

**
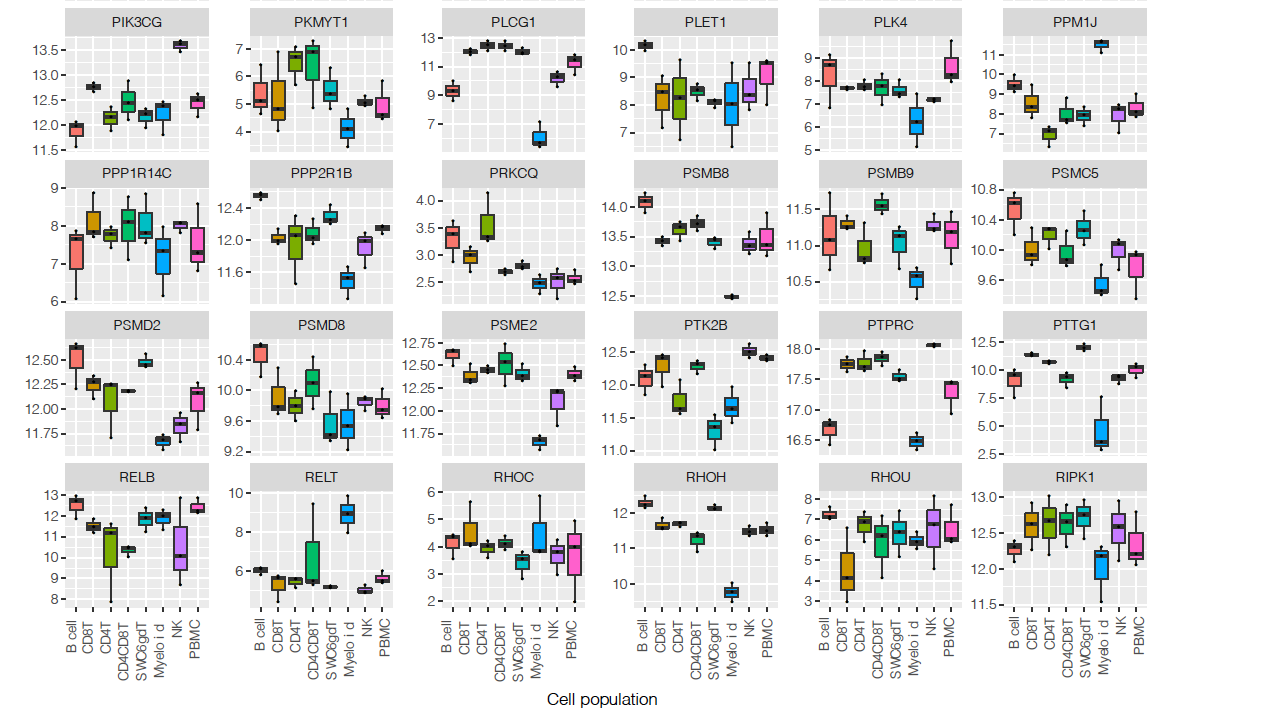
**

**
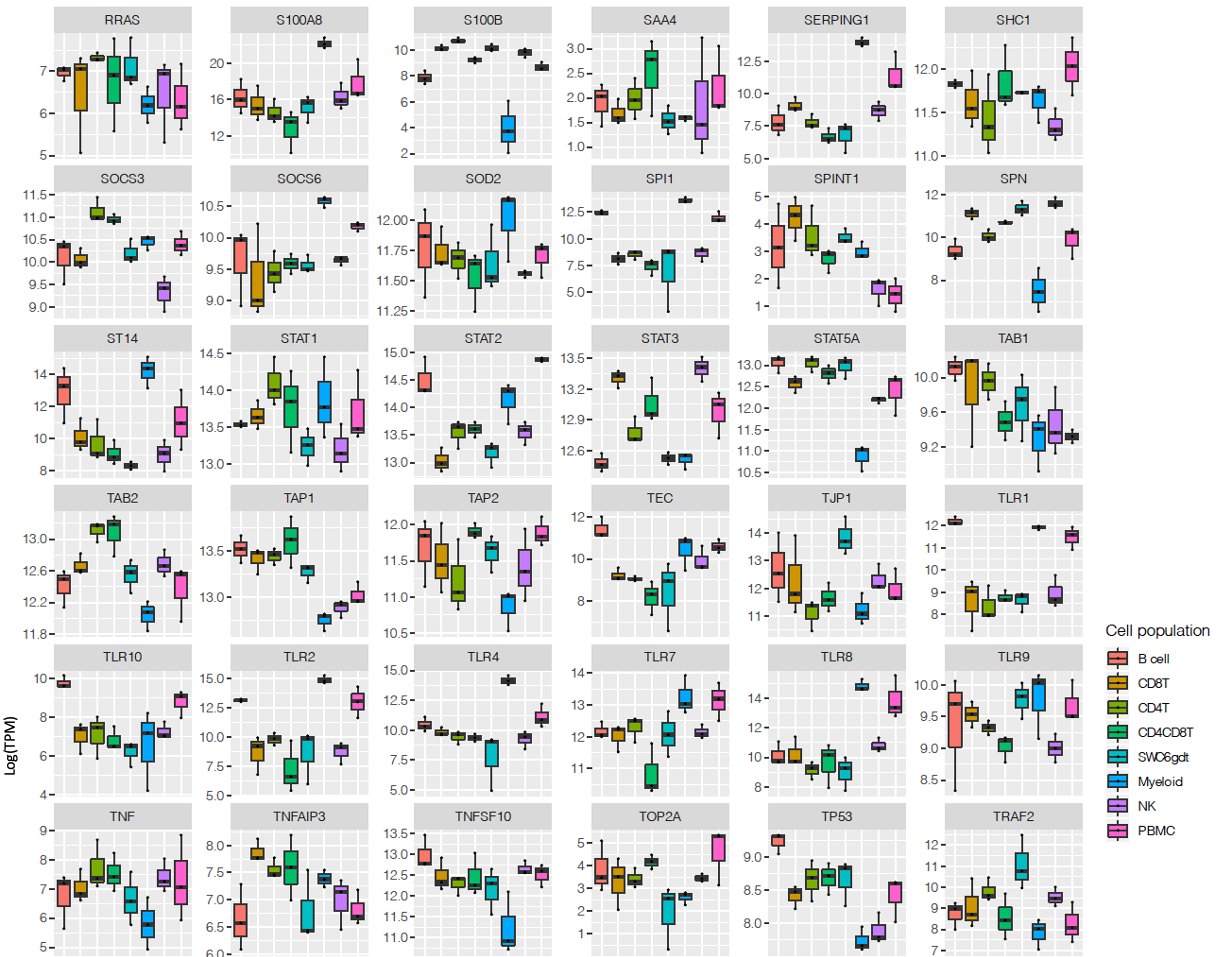
**

**
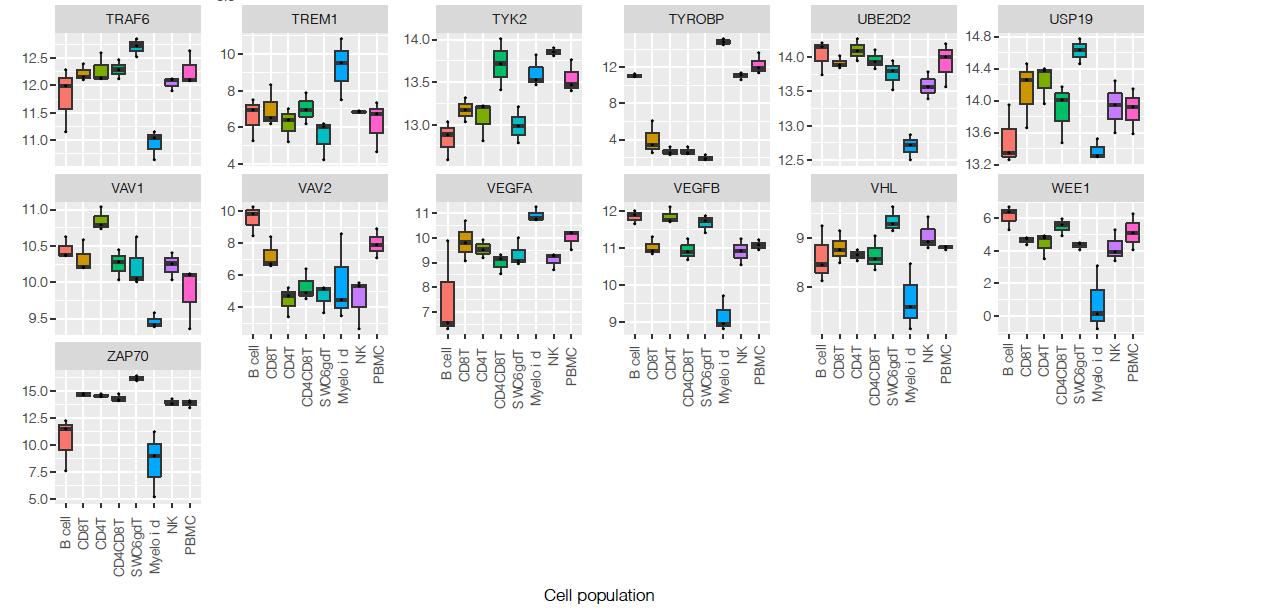
**

**Supplementary Figure 7.** RNA abundance of Nanostring selected genes. Box plot showing log(TPM) expression values of Nanostring selected genes of porcine sorted cells.


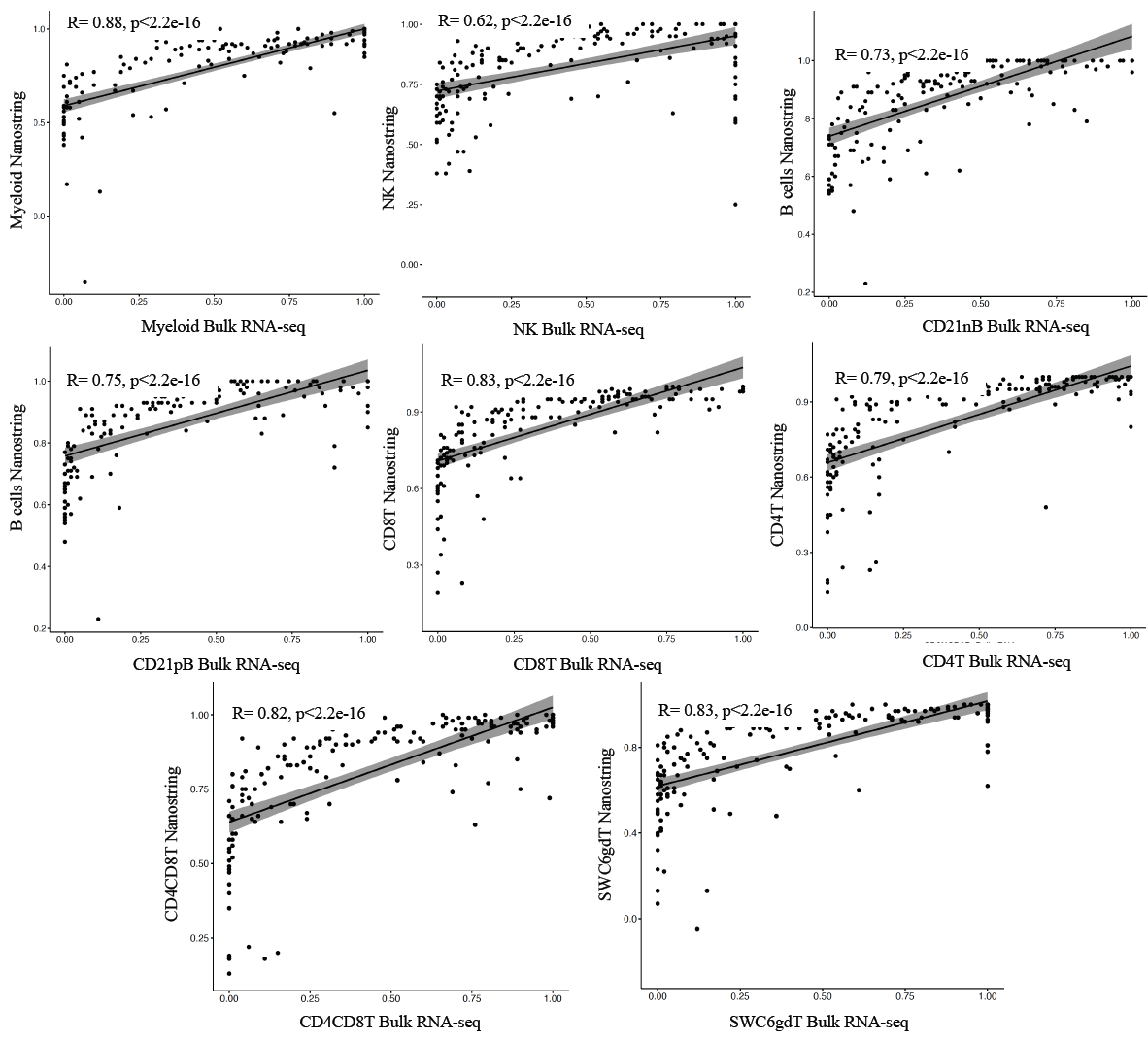


**Supplementary Figure 8.** Bulk RNA and Nanostring correlation. Spearman rank correlation of absolute normalize TPM values of common genes between bulk RNA-seq and Nanostring analysis.

**
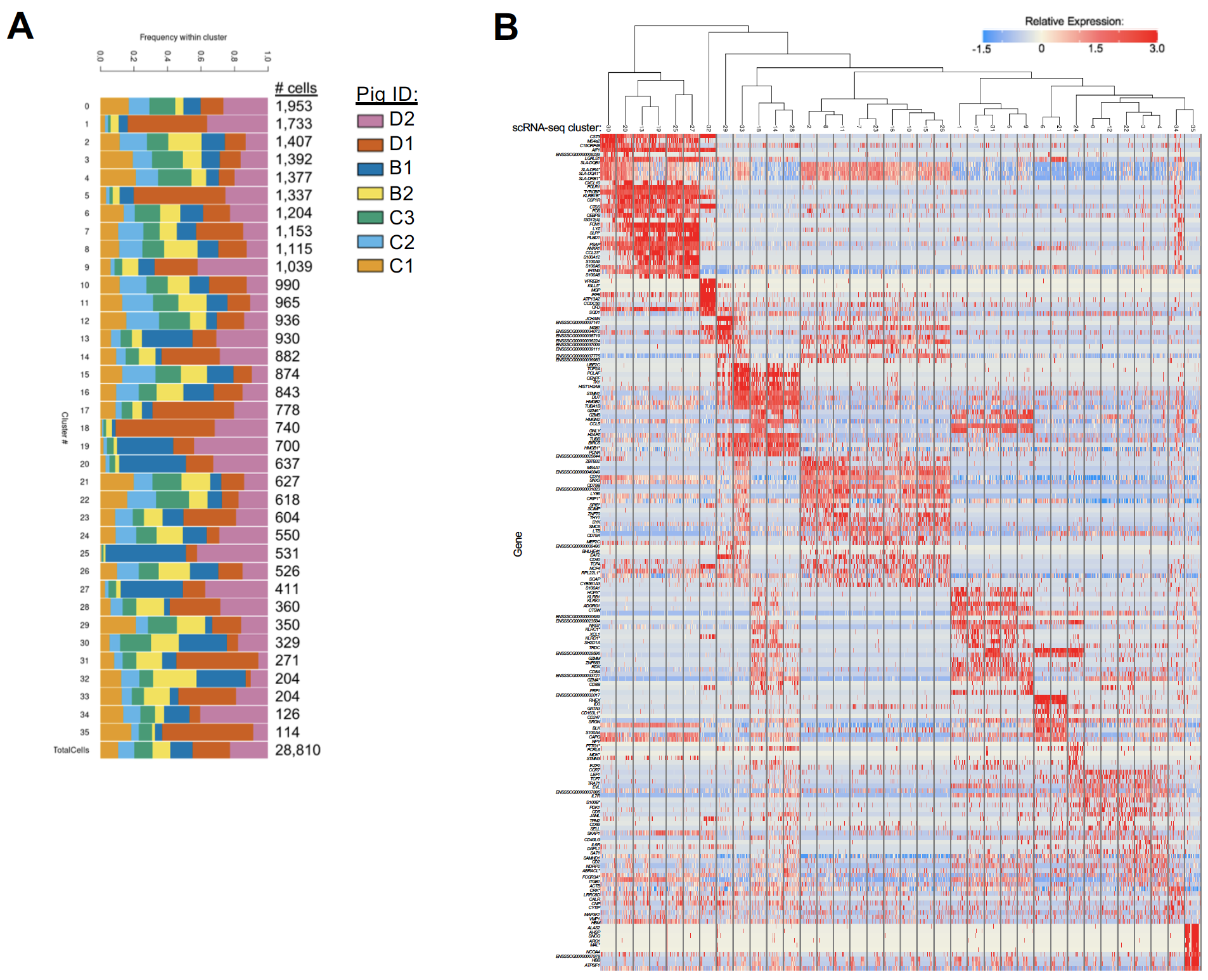
** **
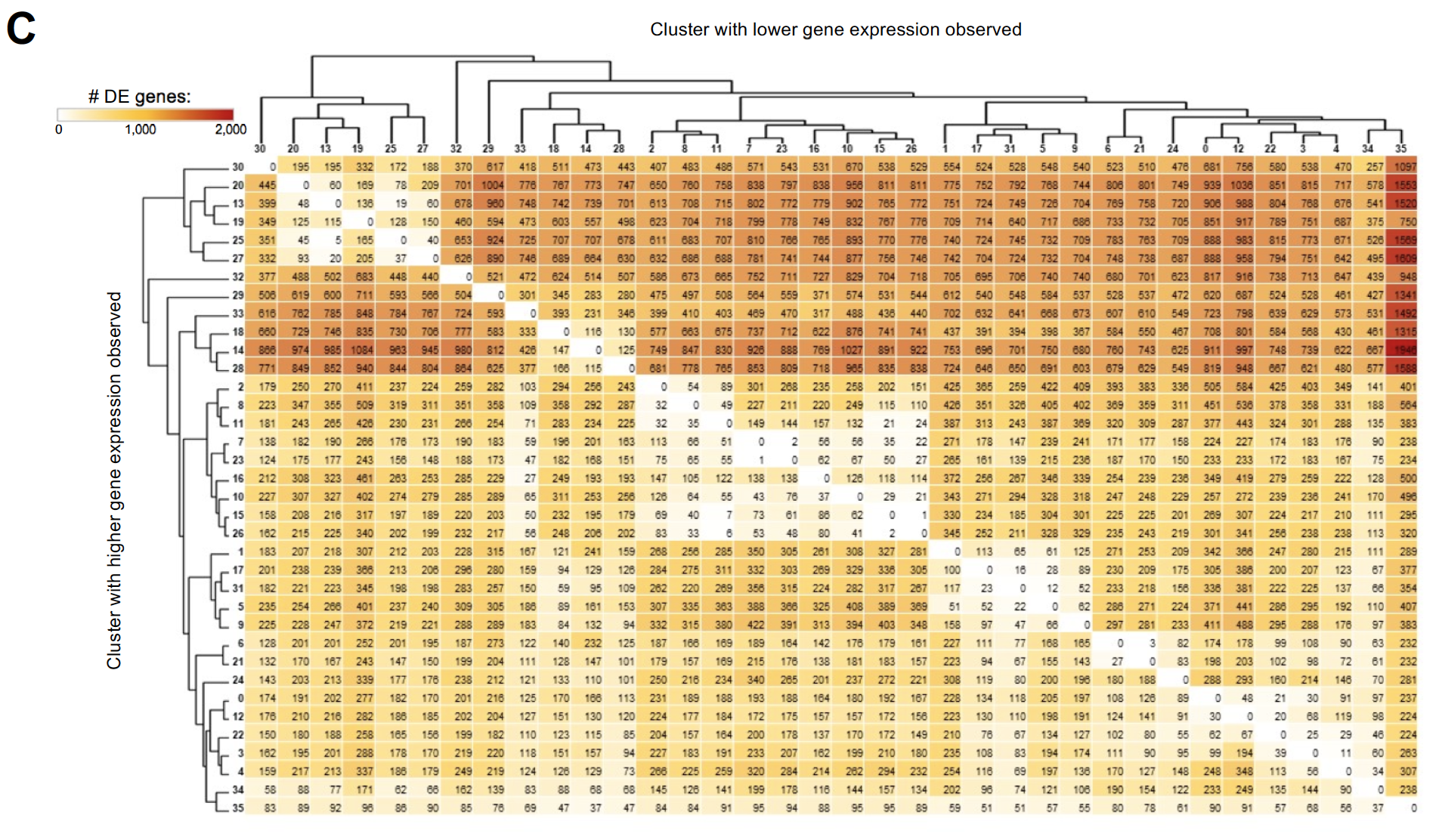
**

**Supplementary Figure 9.** Unique characteristics of scRNA-seq clusters. A) Stacked barplot showing proportion of cells within each cluster derived from different PBMC samples. Bar color corresponds to the animal ID of a sample. Cluster IDs are shown on the left, while the number of cells within each cluster are listed on the right. Proportions of total cells are also listed in the bottom bar. **B)** Heatmap of the top DEGs within scRNA-seq clusters. Clusters are shown on the x-axis, along with a tree showing hierarchical clustering. Genes are listed on the y-axis. Up to the top five differentially expressed genes with highest positive logFC values for each cluster are listed. **C)** Heatmap showing the number of DEGs detected between scRNA-seq clusters by pairwise DGE analyses. Clusters are ordered on the x and y axis according to hierarchical relationships that were also shown in B. The cluster with higher gene expression for a pairwise comparison is shown on the y-axis, while the cluster with lower gene expression by pairwise comparison is shown on the x-axis. Seven PBMC samples used for scRNA-seq analysis were derived from each of three separate experiments (experiment B, n = 2; experiment C, n = 3; experiment D, n = 2) * Refer to ‘Gene name replacement’ methods.


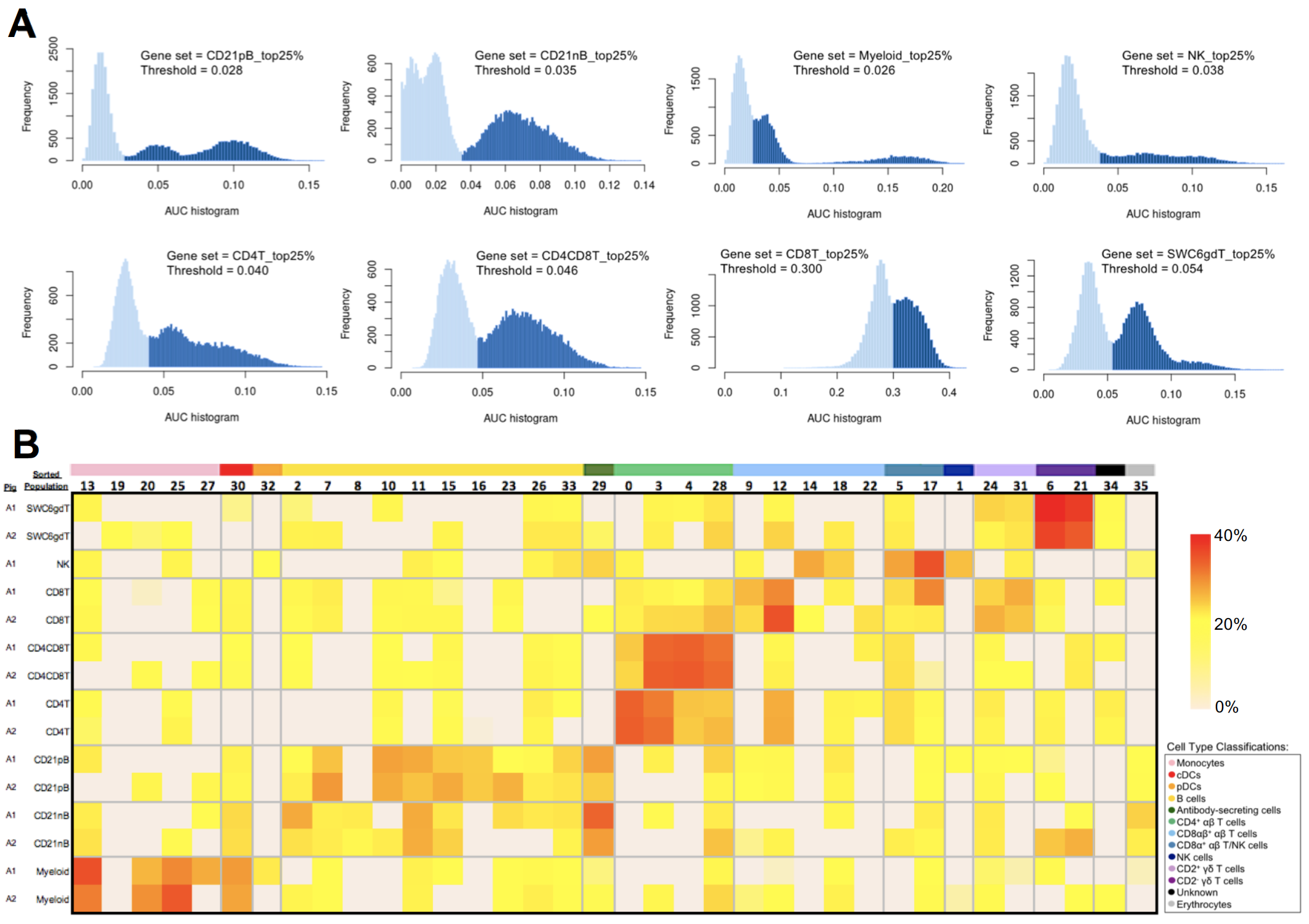

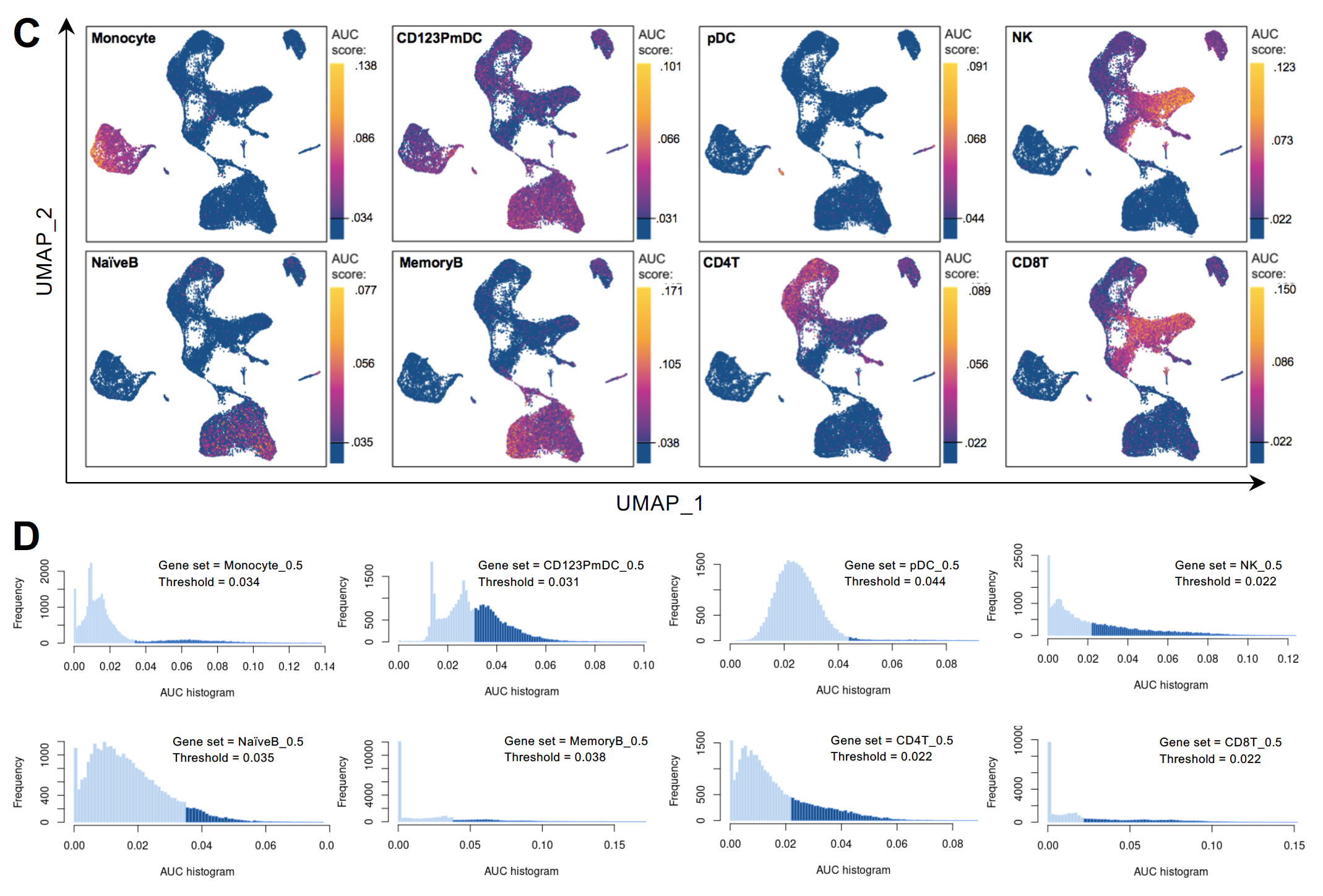


**Supplementary Figure 10.** Additional comparison between bulk and scRNA-seq data. **A)** Histogram of distribution of AUC scores for top 25% gene sets from porcine bulk RNA-seq sorted cell populations depicted in Figure 5 A-B. Frequency (y-axis) corresponds to the number of cells from the porcine scRNA-seq dataset having an AUC score value specified on the x-axis. Light blue bars represent cells falling below a threshold of positive AUC score detection, while dark blue bars represent cells with AUC scores above the threshold of detection used for plots in Figure 5A. Gene set and threshold of detection are provided for each respective histogram. **B)** Heatmap of CIBERSORTx deconvolution results. Porcine sorted cell bulk RNA-seq populations for each animal are listed on the y-axis, and porcine scRNA-seq clusters are listed on the x-axis. A color bar above scRNA-seq cluster IDs on the x-axis indicates the cell type classification, as according to Figure 4D. Heatmap color represents the proportion of bulk RNA-seq cells that correspond to each of the scRNA-seq clusters, with totals across rows for each bulk RNA-seq sample equaling 100%. **C)** Gene set enrichment scores calculated by AUCell analysis of enriched gene sets created from genes with high expression scores > 0.5 for each respective sorted population of human cells overlaid onto cells of the porcine scRNA-seq dataset visualized in 2-dimensional UMAP plot. Each dot represents a single cell. The color of the dot corresponds to the AUC score for each respective cell. Higher AUC scores correspond to greater gene set enrichment. A threshold for AUC score detection was set and indicated by a horizontal line on the gradient fill scale for each plot. The gene set mDC_0.5 was not included due to containing only a single gene used to calculate enrichment scores. **D)** Histogram of distribution of AUC scores from genes with high expression scores > 0.5 for each respective sorted population of human cells depicted in Figure 5 D & Supplementary Figure 10C. Frequency (y-axis) corresponds to the number of cells from the porcine scRNA-seq dataset having an AUC score value specified on the x-axis. Light blue bars represent cells falling below a threshold of positive AUC score detection, while dark blue bars represent cells with AUC scores above the threshold of detection used for plots in Figure 5A. Gene set and threshold of detection are provided for each respective histogram.


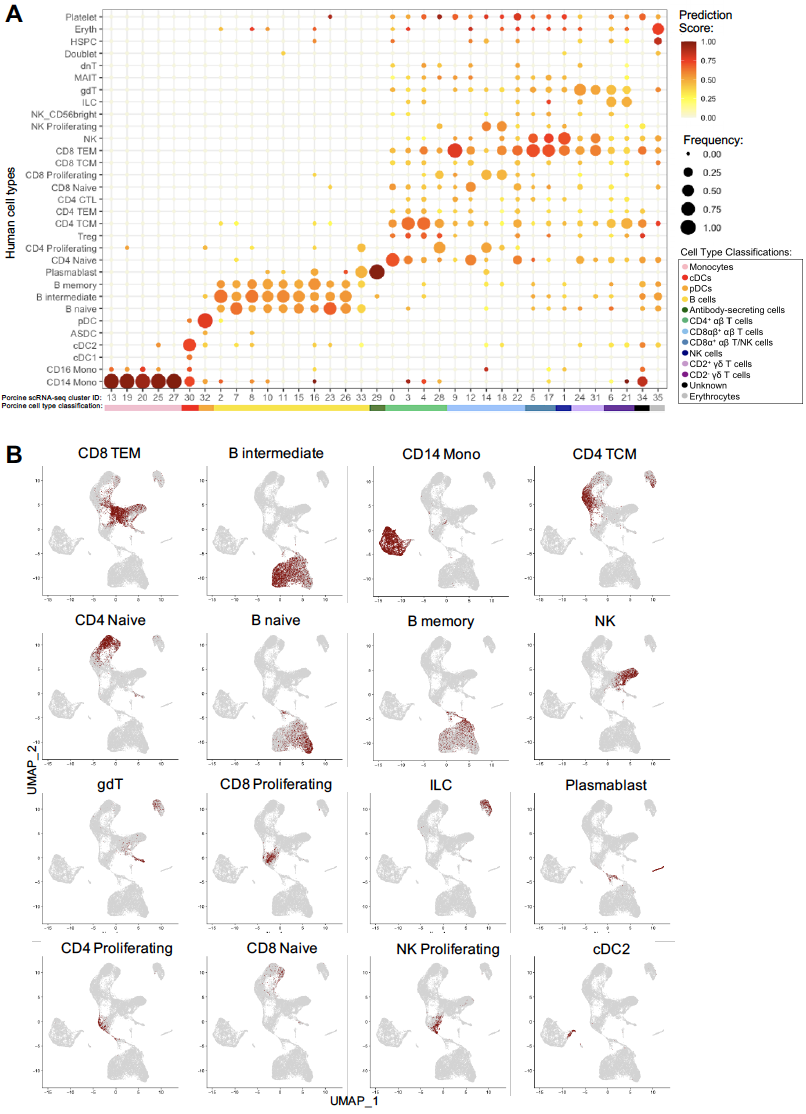


**Supplementary Figure 10.** Integration of porcine and human scRNA-seq datasets and transfer of human cell type labels. **A)** Prediction scores calculated to determine the confidence of cell type annotations. The human cell type specific frequency (size of the circle) and prediction score for that human cell type (color) are shown for each porcine scRNA-seq cluster. Porcine cell type classifications (color) are shown below the porcine scRNA-seq cluster IDs. **B)** The transferred human cell type labels are shown on the original porcine dataset UMAP. Each tile highlights a set of porcine cells annotated with their highest confidence level human cell type label in dark red.
